# From Crisis to Cancer: Diabetic Ketoacidosis Unmasking a Malignancy of Unknown Origin

**DOI:** 10.7759/cureus.99906

**Published:** 2025-12-23

**Authors:** Shadman Sakib Rahman, Nusrat A Chowdhury, Sumaiya Kamal, Muhammad Dalili, Stergios Boussios

**Affiliations:** 1 Internal Medicine, Medway Maritime Hospital, Kent, GBR; 2 Acute Medicine, Medway Maritime Hospital, Kent, GBR; 3 Diabetes and Endocrinology, Medway Maritime Hospital, Kent, GBR; 4 Oncology, Medway Maritime Hospital, Kent, GBR

**Keywords:** acute kidney injury (aki), cancer of unknown primary origin (cup), diabetic ketoacidosis (dka), hyperosmolar hyperglycaemic state, lymphoma, metastasis, tumour markers, whole brain radiation therapy (wbrt)

## Abstract

Diabetic ketoacidosis (DKA) is a life-threatening metabolic emergency, but its presentation as the first sign of an occult malignancy is extremely rare, particularly when the primary site is unknown. We report such a rare case of a 37-year-old woman presenting with progressive headache and vomiting. Initial assessment demonstrated DKA with associated electrolyte disturbances, which were managed promptly, and neuroimaging - prompted by concern for raised intracranial pressure - unexpectedly revealed brain metastases. Despite extensive imaging, immunohistochemistry, and multidisciplinary review, no definitive primary site could be identified. She was diagnosed with cancer of unknown primary (CUP) and referred for whole-brain radiotherapy and palliative care. This case illustrates the rare but critical scenario in which an acute metabolic emergency, such as DKA, unmasks an aggressive metastatic malignancy. The incidental detection of brain metastases in the absence of an identifiable primary tumour adds further diagnostic complexity. Early recognition of red flags, timely imaging, and multidisciplinary coordination are essential for guiding diagnosis and management.

## Introduction

Diabetic ketoacidosis (DKA) is characterised by the presence of hyperglycaemia, ketonaemia (high ketones in blood) and metabolic acidosis that develops very rapidly [1]. DKA is most commonly seen in patients with type 1 diabetes mellitus (DM) but may also occur in patients with type 2 diabetes, particularly in those treated with insulin or sodium-glucose cotransporter-2 (SGLT-2) inhibitors, where mechanisms such as relative insulin deficiency and increased ketogenesis contribute to risk [2]. Other states of hyperglycaemia include DM, hyperglycaemic hyperosmolar state (HHS), impaired glucose tolerance, and stress hyperglycaemia [1].

Very little is known about the relationship between DKA and HHS in patients with cancer [3]. Increased metabolic stress from an advanced cancer can rarely trigger DKA in diabetics and may present as the presenting symptom of an underlying cancer in patients without an obvious cause of DKA but having other red flag signs like weight loss, lymphadenopathy, persistent headaches, etc. [4]. However, it is well known that metastatic spread is a late-onset manifestation in most cancers [5]. Rarely, a few rapidly invasive malignancies can be diagnosed early when metastatic lesions develop without identification of a primary mass [5]. These types of cancers account for 3-5% of all cancers and are referred to as cancer of unknown primary origin (CUP) [5]. The European Society of Medical Oncology (ESMO) and the National Comprehensive Cancer Network (NCCN) guidelines suggest that all patients with metastases secondary to CUP should undergo a thorough physical examination, comprehensive laboratory investigations, and whole-body imaging (CT scan and PET scan) [5]. Age over 60 years is considered one of the weakly linked risk factors that can lead to adenocarcinoma of unknown primary [6].

At present, CUP represents approximately 1-2% of all malignancies, a figure likely reflecting improvements in radiological and molecular diagnostic techniques [7]. Therefore, it is important to include CUP in the differential diagnosis of patients presenting with unexplained DKA, particularly when other red flag features are present, because CUP represents a heterogeneous group of metastatic malignancies that often present atypically and require early comprehensive investigation to guide management and treatment options [8]. Although notable progress has been made, the underlying biological mechanisms of CUP onset and progression are still poorly understood, raising concerns about the completeness and accuracy of the diagnostic evaluation at each presentation [9]. Molecularly directed therapies represent a new therapeutic era in the management of patients with CUP [10].

## Case presentation

We report the case of a 37-year-old South Indian woman with a background of type 2 DM complicated by microvascular disease and uterine leiomyoma. She presented to the emergency department with persistent nausea and multiple episodes of vomiting. She reported reduced tolerance of solid food and had been relying predominantly on fluids such as water and tea. She also described an unintentional weight loss of approximately 7 kg over the preceding three months. She denied night sweats or respiratory symptoms but reported travel to Sri Lanka one year earlier. Two days before presentation, she had been commenced on oral antibiotics by her primary care physician for presumed urinary tract infection following new-onset urinary urgency and frequency.

On arrival, she appeared clinically dehydrated but remained haemodynamically stable. Venous blood gas analysis demonstrated severe metabolic acidosis, hyperglycaemia, and elevated ketone levels, consistent with DKA. Routine laboratory investigations revealed mildly elevated inflammatory markers, hypomagnesaemia, and hypophosphataemia (Table 1). Capillary blood glucose was 15.2 mmol/L. Urinalysis showed 2+ glucosuria, trace blood, and negative ketones at the time of testing. Physical examination revealed mild suprapubic tenderness, clear lung fields, and no focal neurological deficits. Further examination revealed enlarged lymph nodes in the right submandibular area and the anterior left cervical chain.

**Table 1:** Routine blood investigations.

Investigations (with units)	Results	Normal values
Prothrombin time (seconds)	15.7	9.4-12.5
International normalised ratio (ratio)	1.4	0.8-1.2
Activated partial thromboplastin time (APTT) (seconds)	35.7	25.1-36.5
Amylase (IU/L)	53	28-100
Calcium (mmol/L)	2.48	2.20-2.60
Adjusted calcium (mmol/L)	2.46	2.20-2.60
Phosphate (mmol/L)	0.65	0.80-1.50
Magnesium (mmol/L)	0.65	0.70-1.00
Albumin (g/L)	41	35-50
Alkaline phosphatase (ALP) (U/L)	106	30-130
Alanine aminotransferase (ALT) (U/L)	16	<35
Total bilirubin (µmol/L)	7	0-21
Urea (mmol/L)	2.9	2.5-7.8
Creatinine (µmol/L)	48	45-84
Estimated GFR (mL/min/1.73 m^2^)	>90	>60
Sodium (mmol/L)	133	133-146
Potassium (mmol/L)	4.1	3.5-5.3
High-sensitivity CRP (mg/L)	26.0	0.0-5.0
White blood cell count (×10^9^/L)	9.1	4.0-11.0
Red blood cell count (×10^12^/L)	4.72	3.80-4.80
Haemoglobin (g/L)	132	120-150
Haematocrit (L/L)	0.39	0.36-0.46
Mean cell volume (MCV) (fL)	83.4	80.0-100.0
Platelet count (×10^9^/L)	503	150-410
Neutrophils (×10^9^/L)	4.6	2.0-7.0
Lymphocytes (×10^9^/L)	3.8	1.0-4.0
Glucose (mmol/L)	15.2	3.5-5.4

She was commenced on DKA management, including fixed-rate intravenous insulin infusion, intravenous fluid resuscitation, and electrolyte replacement. Empirical antibiotics were continued for a suspected urinary tract infection. Her metabolic acidosis improved with treatment, and repeat venous blood gas confirmed resolution of DKA (Table 2).

**Table 2:** Serial venous blood gas (VBG) measurements during the diagnosis and management of diabetic ketoacidosis (DKA). Initial VBG was obtained at the time of DKA diagnosis. “During treatment” reflects values after fluid and insulin therapy, and “At DKA resolution” represents near-normalisation of metabolic parameters following treatment.

Parameter (units)	Initial (on DKA diagnosis)	During treatment	Post-treatment (DKA resolution)	Normal values
pH	7.18	7.30	7.38	7.35-7.45
pCO_2_ (kPa)	5.2	5.1	4.0	4.7-6.0 kPa
Sodium (Na⁺, mmol/L)	132	133	133	135-145 mmol/L
Potassium (K⁺, mmol/L)	3.9	4.6	3.7	3.5-5.0 mmol/L
Glucose (mmol/L)	18.2	12.7	15.1	3.5-7.8 mmol/L
Lactate (mmol/L)	1.9	1.7	1.2	0.5-2.2 mmol/L
Base excess (mmol/L)	-13.8	-7.4	-8.0	-2 to +2 mmol/L
Bicarbonate (HCO_3_^-^, mmol/L)	13.0	18.1	19.4	22-28 mmol/L
Ketones (mmol/L)	5.0	1.1	0.2	<0.6 mmol/L

After being transferred to the acute medical ward, during further assessments, she reported a three-week history of a progressively worsening frontal headache, described as dull in character and worse in the morning. The headache had persisted despite regular paracetamol use. Given the persistence of symptoms despite metabolic stabilisation and concern for raised intracranial pressure, an urgent CT scan of the head was requested.

**Figure 1:**
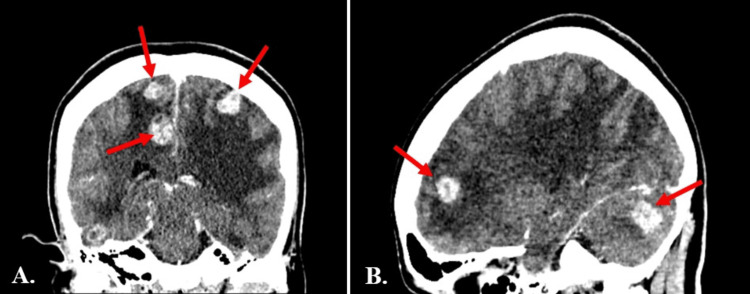
Contrast-enhanced computed tomography of the brain showing (A) coronal and (B) sagittal sections demonstrating multiple enhancing intracranial lesions with surrounding vasogenic oedema and moderate mass effect (arrows).

The findings observed on the CT scan were quite unexpected. The scan showed multiple enhancing mass lesions in the parenchyma with significant surrounding vasogenic oedema, resulting in moderate mass effect (Figure 1). The case was discussed urgently with the neurosurgical team, who advised that there was no acute surgical intervention required at this stage and recommended conservative management. They further advised arranging an urgent MRI of the brain and a CT scan of the thorax, abdomen, and pelvis to evaluate for a possible primary source of the intracranial lesions.

**Figure 2:**
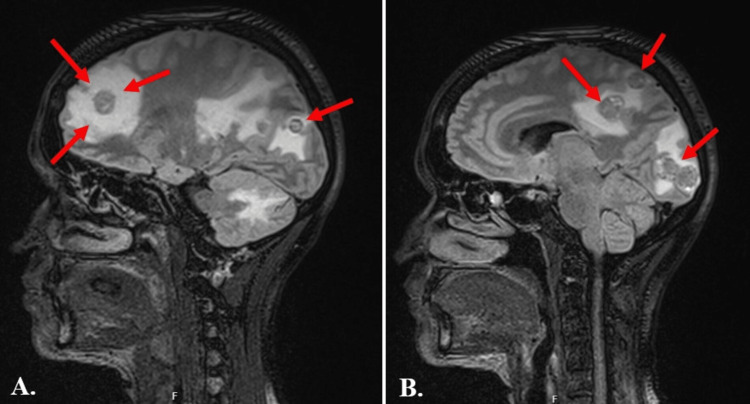
Contrast-enhanced magnetic resonance imaging of the brain (sagittal sections) showing multiple heterogeneously enhancing metastatic lesions with surrounding vasogenic oedema. (A) Lesions involving the frontal and occipital lobes (arrows). (B) Lesions involving the temporal, parietal, and occipital lobes with associated mass effect (arrows).

MRI brain demonstrated haemorrhagic nodular enhancing lesions with surrounding oedema and a 5 mm rightward midline shift (Figure 2). The radiological appearance raised strong suspicion for metastatic disease, with a differential diagnosis including high-grade lymphoma and granulomatous disease. Infectious screen, including HIV, TB and *Toxoplasma* screen was done, which came back negative (Table 3). A panel of serum tumour markers were also done (Table 4).

**Table 3:** Results of the infectious screening panel. HIV: human immunodeficiency virus; TB IGRA: tuberculosis interferon gamma release assay

Test	Results
*Toxoplasma* screen	IgG NOT detected; IgM NOT detected
HIV	NOT detected
Hepatitis B surface antigen (HBsAg)	NOT detected
Hepatitis C antibody	NOT detected
Tuberculosis test (TB IGRA)	NOT detected

**Table 4:** Results of tumour marker assessment. CA: cancer antigen; β-hCG: beta human chorionic gonadotropin

Tumour markers with units	Results	Normal value
Alpha-fetoprotein (AFP) (KU/L)	1.0	<7.4
CA-125 (KU/L)	82	<35
Serum β-hCG (IU/L)	2.0	0.0-5.0
Serum LDH (IU/L)	2267	120-250

**Figure 3:**
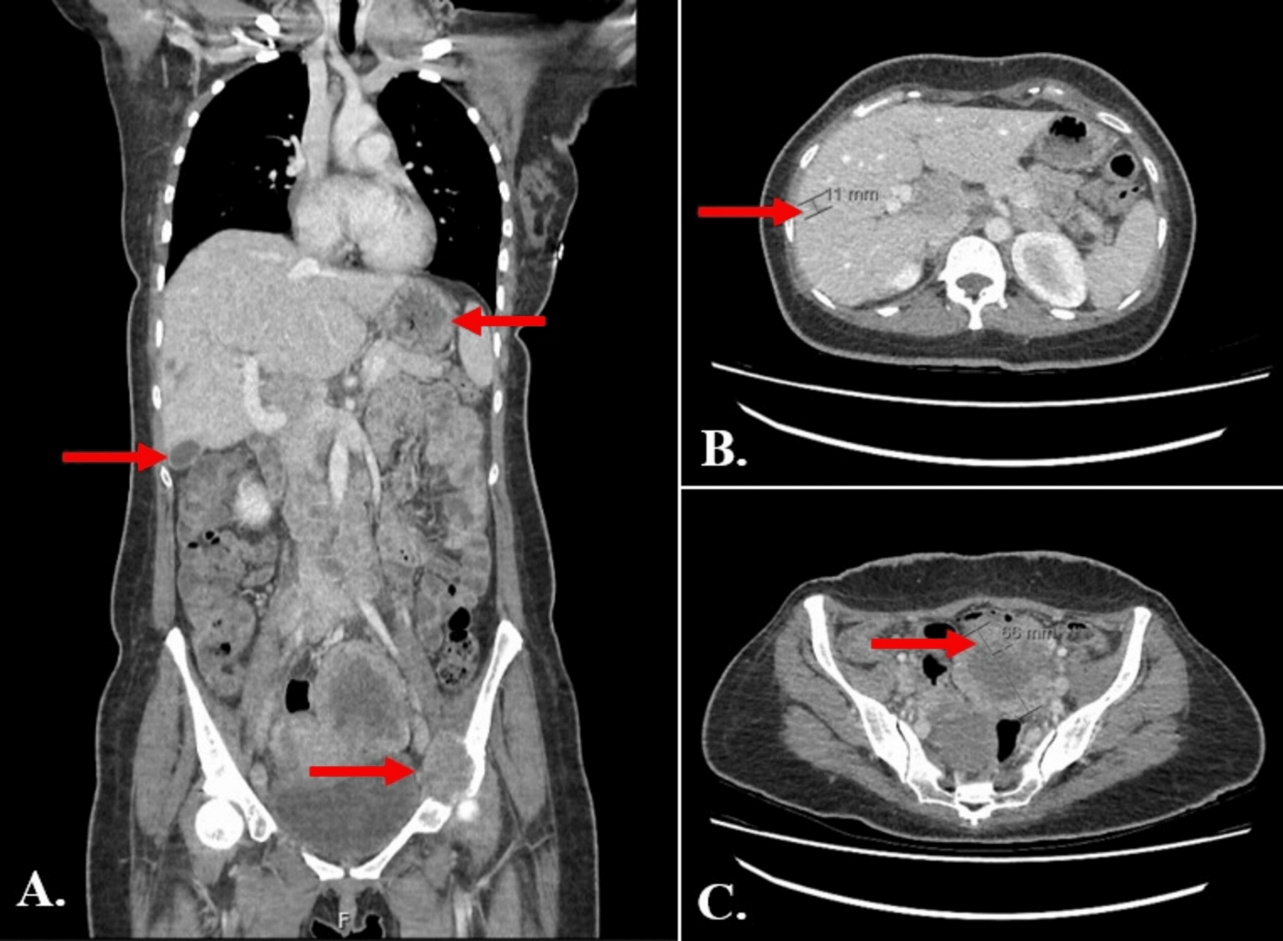
(A) Contrast-enhanced computed tomography of the thorax, abdomen, and pelvis (TAP), coronal section, showing extensive lymphadenopathy, hypoattenuating hepatic lesions, a complex left ovarian cystic mass, and a lytic lesion involving the left acetabular roof (arrows). (B-C) Axial sections demonstrating a small hypoattenuating hepatic lesion (11 mm; arrow) and a left ovarian cyst (6.6 cm) with nodular enhancing components (arrow).

Further systemic imaging with CT thorax, abdomen, and pelvis revealed widespread lymphadenopathy in cervical, mediastinal, retroperitoneal, pelvic, and inguinal regions. Additionally, there were bilateral cystic ovarian lesions described as “aggressive looking” and a few small hypoattenuating lesions in the liver (Figure 3).

**Figure 4:**
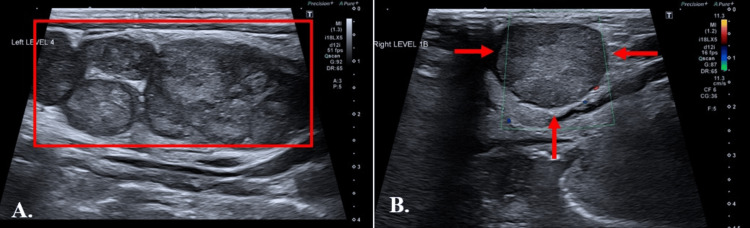
Ultrasound of the neck showing (A) multiple enlarged, heterogeneous cervical lymph nodes arranged in a matted cluster (boxed) and (B) an enlarged right submandibular lymph node (arrows).

An ultrasound (US) neck was performed, which stated enlarged and matted lymph nodes in the left cervical chain and submandibular region (Figure 4).

**Figure 5:**
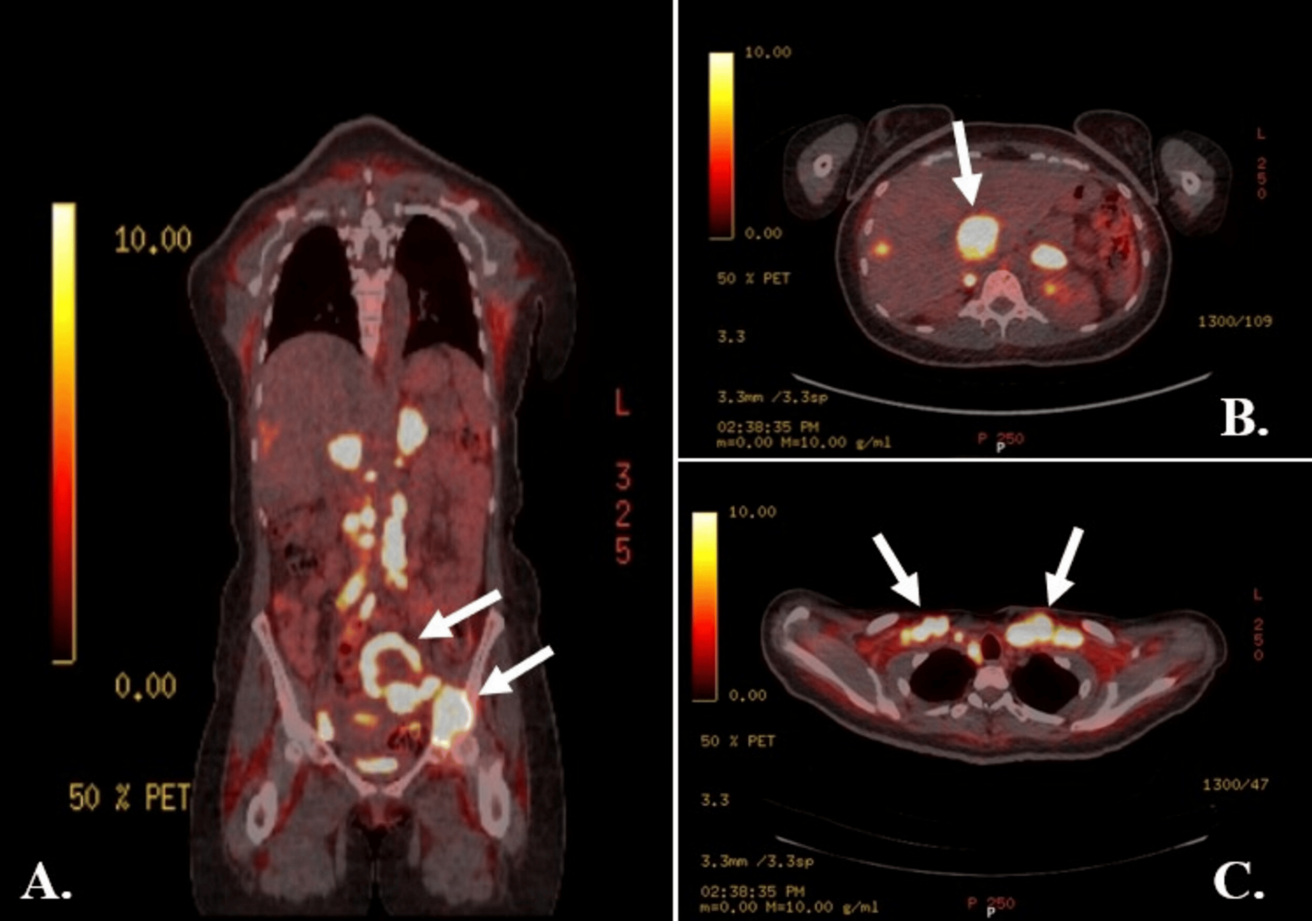
PET-CT images demonstrating disseminated metastatic disease. (A) Coronal section showing a large hypermetabolic left pelvic mass with extensive FDG-avid lymphadenopathy above and below the diaphragm, and involvement of the left acetabulum (arrows). (B) Axial section showing an FDG-avid metastatic deposit in the right hepatic lobe (arrow). (C) Axial section demonstrating bilateral mediastinal lymphadenopathy (arrows). PET-CT: positron emission tomography-computed tomography; FDG: fluorodeoxyglucose

PET-CT demonstrated a large, partially necrotic hypermetabolic mass in the left pelvis, with extensive hypermetabolic lymphadenopathy above and below the diaphragm and associated bony erosion of the left acetabulum (Figure 5). Additional distant metastases were identified, including hepatic and adrenal lesions, pulmonary deposits, and a subcutaneous occipital nodule.

**Figure 6:**
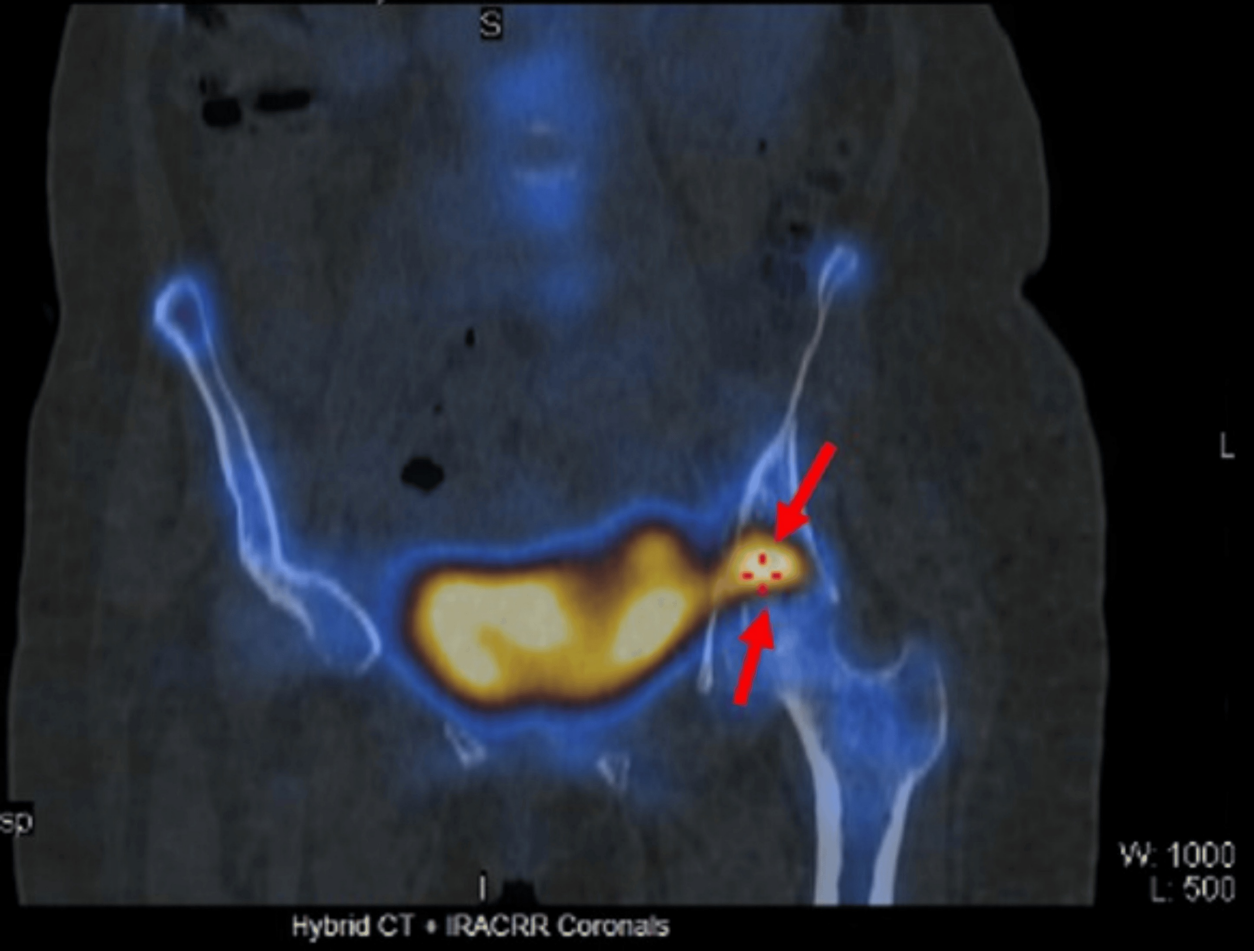
NM bone scan (coronal view) demonstrating a large osteoblastic metastatic lesion in the left acetabulum (arrows). NM bone scan: nuclear medicine bone scan (also known as a bone scintigraphy)

Nuclear medicine bone scan demonstrated a large osteoblastic metastatic lesion in the left acetabulum with no other osteoblastic skeletal metastases (Figure 6).

The constellation of imaging findings as well as blood results pointed to two main working diagnoses: either a metastatic gynaecological malignancy, likely of ovarian origin, or a lymphoproliferative disorder such as lymphoma. CA-125 was moderately raised at 82 KU/L. A lymph node biopsy from the submandibular region was performed, and the report was discussed within the multidisciplinary team (MDT) (Table 5).

**Table 5:** Summary of right submandibular lymph node biopsy results. BerEP4: Ber-Epithelium antigen 4; EMA: epithelial membrane antigen; CK: cytokeratin; p40: ΔNp63 isoform (squamous marker); CD56: neural cell adhesion molecule; CDX2: caudal-type homeobox 2; WT1: Wilms tumour protein 1; TTF-1: thyroid transcription factor-1; GATA3: GATA binding protein 3; ER: estrogen receptor; p63: tumour protein p63; Napsin A: aspartic protease napsin A; CUP: cancer of unknown primary; MDM: multidisciplinary meeting

Specimen	Right submandibular lymph node
Clinical details	Histology right submandibular pathological lymph node core biopsy.
Macroscopic examination	Two cores and multiple fragments are split across two cassettes. One core and a fragment in A1 and one core in A2. The largest core measures 10mm.
Microscopic examination	Cores of fibrovascular tissue and skeletal muscle, the former showing extensive infiltration by a partly necrotic tumour composed of nests and islands of discohesive malignant epithelioid cells. In some areas, dirty necrosis is apparent. Some squamoid features are noted focally. Immunohistochemistry is in progress in order to determine the likely site of primary of this poorly differentiated carcinoma, and a further report will follow.
Supplementary report	Immunohistochemistry is strongly and diffusely positive at BerEP4 and focally positive for EMA. There is also focal positivity for CK7, and a small number of cells are positive for p40 in the more squamoid appearing areas. The tumour cells are negative for CK20, CK56, CD56, CX2, WT1, TTF-1, GATA3, ER, p63 and NAPSIN. This immuno-profile is non-specific, with strong BerEP4 positivity supporting a diagnosis of metastatic adenocarcinoma despite some squamoid features and focal positivity for p40. Negativity for GATA3, CDX2 and TTF1 napsin makes lung, lower gastrointestinal tract and breast origin unlikely, although not entirely excluded. Correlation with imaging, clinical history and serum tumour marker studies is recommended with discussion in the CUP MDM.

Management

While the patient was being treated as per DKA protocol, she was simultaneously started on levetiracetam as seizure prophylaxis due to a significant mass effect seen on the images of her brain. High-dose intravenous dexamethasone was started after receiving the biopsy report, to reduce the surrounding vasogenic oedema along with appropriate proton pump inhibitor (PPI) cover.

Clinically, her nausea settled, and she tolerated oral intake, but she continued to experience persistent headache, back pain, and discomfort in her legs and abdomen. Her pain was managed with regular paracetamol, modified-release oral morphine at night, and additional oral morphine as required.

Since lymphoma was excluded on core biopsy with histopathology and immunohistochemical analysis, the case was reviewed by the gynaecology MDT in view of the pelvic mass. Based on the overall clinical features, blood results, imaging findings, and biopsy report, the MDT concluded that the disease was not of gynaecological origin, and the patient was therefore referred through the CUP pathway for further investigation and management (Table 6).

**Table 6:** Outcome of the gynaecological multidisciplinary team discussion. EMA: epithelial membrane antigen; p53: tumour protein p53; PAX8: paired box gene 8; p16: cyclin-dependent kinase inhibitor 2A; OCT3/4: octamer-binding transcription factor 3/4; MDM: multidisciplinary meeting

Gynaecological MDM	Outcome
Conclusion	There is agreement on a poorly differentiated carcinoma of uncertain primary origin. However, EMA staining was regarded as marking plasma cells rather than tumour cells. Additional immunohistochemistry performed demonstrated wild-type p53 staining; virtually negative staining for PAX8, p16 and negativity for OCT3/4. The appearances are not typical for a primary gynaecological malignancy, and indeed, in addition to the ovarian masses, there is widespread metastatic disease such that the ovarian tumour is probably also metastatic. The tumour morphology is also not typical of a malignant germ cell tumour.

Her case was also referred to the neuro-oncology team for MDT discussion, and they advised that she was not a suitable candidate for surgical treatment or stereotactic radiosurgery (SRS) and to consider whole-brain radiation therapy (WBRT).

The patient was discharged in a clinically stable condition with ongoing follow-up planned under the acute oncology team and CUP team in the community. She was due to start her radiotherapy and palliative chemotherapy if required.

## Discussion

DKA is an acute metabolic emergency that is life-threatening and requires urgent intervention for a favourable outcome [1]. There are multiple stressors for this condition, but CUP has not been established as one of them.

CUP is known to manifest very aggressively and spreads very rapidly [11]. Investigations to determine the primary site include comprehensive histopathological analysis along with targeted immunohistochemistry and radiological evaluation [11]. Based on their prognosis, CUP patients are divided into favourable (20%) and unfavourable (80%) groups, with systemic platinum-based chemotherapy being offered to the former and empirical chemotherapy based on combination regimens of platinum or taxane to the latter [11]. However, response to therapy and chances of survival are poor in the unfavourable subset [11].

A systematic review involving 884 patients with CUP, based on 12 autopsy cohort studies conducted from 1944 to 2000, found that autopsy was able to identify the primary tumour site in 644 cases (73%) [12]. The most frequently identified primary sites were the lung (27%), pancreas (24%), hepatobiliary system (8%), kidneys (8%), bowel (7%), genitourinary tract (7%), and stomach (6%) [12]. 

Similar cases have been documented in various literature as described in Table 7.

**Table 7:** Summary of similar reported cases. CNS: central nervous system; R-CHOP: rituximab, cyclophosphamide, hydroxydaunorubicin (doxorubicin), oncovin (vincristine), and prednisolone; OC: ovarian cancer; DKA: diabetic ketoacidosis; CA: cancer antigen

Author/year	Age/sex	Diagnosis	Presentation	Key features	Outcome
Kahn et al. (2020) [13]	34F	Epithelial ovarian carcinoma	Brain metastasis at diagnosis	Metastasis to Meckel’s cave, leptomeningeal spread	Rapid clinical decline; palliative care initiated
Costello et al. (2023) [14]	Various	Epithelial ovarian carcinoma	Brain metastases (review/case series)	Incidence ~1-3% of OC cases; usually a late manifestation	Median survival is 6-12 months after CNS involvement
Huang et al. (2022) [15]	47F	Diffuse large B-cell lymphoma (DLBCL) involving the ovary	Ovarian mass, widespread lymphadenopathy	CA-125 elevated; mimicked ovarian carcinoma	Responded to R-CHOP chemotherapy
Le et al. (2024) [16]	56M	Pancreatic adenocarcinoma	DKA is the first manifestation	Illustrates malignancy presenting with metabolic crisis	Rapid progression; poor survival

Owing to the heterogeneity of CUP, it can manifest as a wide array of clinical features depending on the site of malignant involvement [12]. The inability to determine the primary site during the lifetime of a patient poses a significant challenge to clearly establish diagnosis and treatment, which creates a difficult situation for the patient as well as the healthcare providers [9].

**Figure 7:**
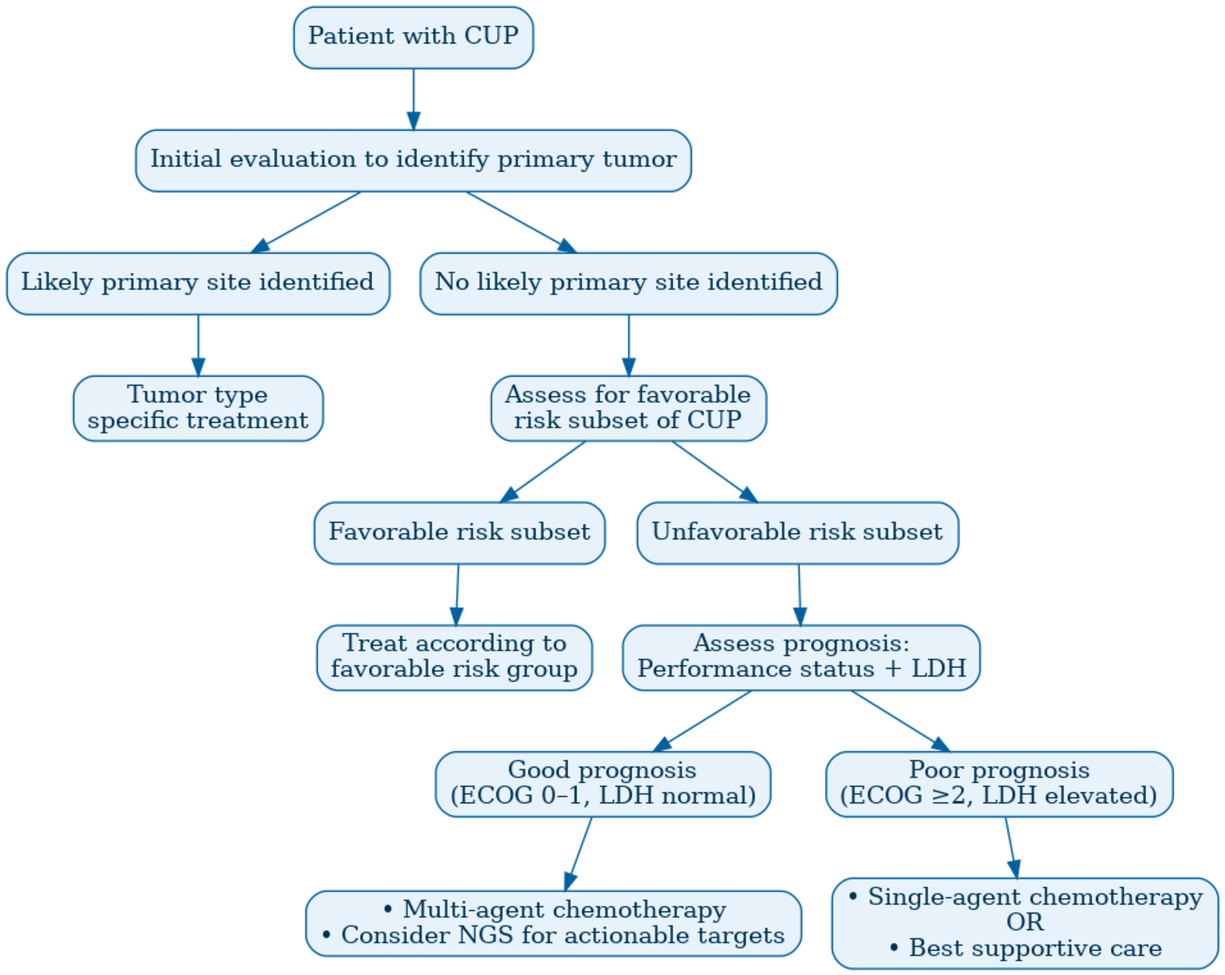
Diagnostic and treatment pathway for CUP, adapted from the European Society for Medical Oncology (ESMO) and the National Comprehensive Cancer Network (NCCN) guidelines. ECOG: Eastern Cooperative Oncology Group; LDH: lactate dehydrogenase; CUP: cancer of unknown primary This image has been recreated, while preserving the structure and content of the original figure published in Reference [12]. Image credit: Nusrat Ahmed Chowdhury, adapted from Michael S. Lee, 2020 [12]

Following histopathological evaluation, if the primary site remains undiscovered, further investigation should be led by consensus guidelines developed by leading cancer agencies, as shown in Figure 7 [12].

Patients with DM are found to have elevated tumour markers, but their role in DKA and HHS is not known [17]. The prognosis of adenocarcinoma, if left untreated, is poor, and the median survival ranges between three and four months [6]. Therefore, the main aim of diagnostic investigations should be to locate the subtypes that are more likely to benefit from any active treatment [6].

Most of these patients are resistant to chemotherapy and receive an empirical regimen, but with constrained longevity [18]. Nevertheless, CUP is still considered life-threatening and is identified as among the seventh or eighth leading cause of mortality among cancer patients [18]. These tumours may respond well to immune checkpoint inhibitors (ICI) by triggering a stronger anti-tumour immune reaction [19].

## Conclusions

It is quite rare to encounter a patient presenting with DKA as an initial symptom for an occult malignancy, and that too with an unknown primary. Another uncommon feature is the incidental findings of brain metastasis as a first sign in the absence of a primary tumour. Both conditions were present in this young lady, which makes her case extremely unique. This case highlights how incidental discovery of metastatic brain lesions underscores how acute metabolic crises can unmask serious underlying pathologies, including malignancies of unknown origin. The two main differentials were high-grade lymphoma or an ovarian malignancy, both of which have been excluded after a full panel of investigations, and the working diagnosis remains CUP. The treatment options that remain now are empirical treatment, the nature of which is highly supportive and not curative, along with comprehensive and engaging communication with the patient to support her in every way possible.

It can be challenging to complete an entire diagnostic workup for such patients in an acute setting and to find the right balance between stabilising the patient while simultaneously carrying out the cancer evaluation. In such complex conditions, a multidisciplinary collaboration is essential to consider broader differentials and initiate timely investigations in the presence of clinical red flags.
